# Depletion of CD169^+^ border-associated macrophages induces Parkinson’s disease-like behavior

**DOI:** 10.3389/fnins.2025.1688394

**Published:** 2025-12-04

**Authors:** Takuto Ohki, Kai Kitamura, Katsuhiro Tokutake, Sota Saeki, Michiro Yamamoto, Masaki Takasu, Hitoshi Hirata

**Affiliations:** 1 Department of Hand Surgery, Nagoya University School of Medicine, Nagoya, Japan; 2 Department of Veterinary Medicine, Faculty Applied Biological Sciences, Gifu University, Gifu, Japan; 3 Institute for Advanced Study, Gifu University, Gifu, Japan; 4 Center for One Medicine Innovative Translational Research (COMIT), Gifu University, Gifu, Japan; 5 School of Medicine, Dentistry and Pharmaceutical Sciences, Okayama University, Okayama, Japan

**Keywords:** neuroimmunologic disorders, Parkinson’s disease, macrophage, innate immune system, olfactory bulb

## Abstract

Parkinson’s disease (PD) and Alzheimer’s disease (AD) present with complex behavioral symptoms that can arise in the absence of overt structural brain damage. Recent evidence suggests that border-associated macrophages (BAMs) located at the brain’s interfaces regulate central nervous system function, yet the specific roles of distinct BAM subsets remain largely undefined. By reanalyzing single-nucleus RNA sequencing data from postmortem PD brains, we identified a BAM subset expressing CD169 that was significantly reduced in patients compared with controls. To examine their function, we employed CD169-DTR mice to selectively ablate CD169^+^ BAMs and evaluated behavioral and histological changes. Depletion of CD169^+^ BAMs induced tremors, abnormal hindlimb reflexes, and heightened anxiety-like behavior without dopaminergic neuron loss. Histological analysis revealed a pronounced reduction of mitral and tufted cells in the olfactory bulb, indicating disruption of olfactory-limbic circuitry. These findings demonstrate that CD169^+^ BAMs are critical for maintaining neural network stability and motor function, and that their loss can elicit PD-like phenotypes in the absence of classical dopaminergic neurodegeneration. This work establishes a novel mouse model linking brain-border immune cell dysfunction to Parkinsonian pathology and highlights a neuroimmune mechanism that may contribute to the onset of PD-like disorders.

## Introduction

Parkinson’s disease (PD) and Alzheimer’s disease (AD) are common and debilitating neurodegenerative disorders characterized by motor and non-motor symptoms that can arise in the absence of overt structural brain damage (Prell, 2018; Frisoni et al., 2025). While the pathological hallmarks of these conditions, such as dopaminergic neurodegeneration in PD and amyloid/tau pathology in AD, have been well described, accumulating evidence indicates that early disease stages involve subtle circuit-level dysfunction before substantial neuronal loss occurs (McMackin et al., 2019; Yau et al., 2018). Understanding the mechanisms that destabilize neural networks in the prodromal or early phases of disease is therefore critical for elucidating disease pathogenesis and identifying novel therapeutic targets.

In recent years, the brain’s border regions—including the meninges, perivascular spaces, and choroid plexus—have garnered attention as critical sites of neuroimmune interaction. These interfaces are populated by specialized immune cells known as border-associated macrophages (BAMs), which play important roles in immune surveillance, vascular homeostasis, and communication with the central nervous system (CNS) (Schonhoff et al., 2023; Vara-Perez and Movahedi, 2025; Silvin et al., 2023). However, the functional diversity of BAM subsets and their impact on CNS activities and behavior remain largely unexplored. CD169 (also known as Siglec-1) is a sialic acid-binding receptor expressed by a subset of BAMs (Silvin et al., 2023). Previous studies have suggested that CD169^+^ macrophages exhibit unique phenotypic and spatial characteristics, but their relevance to human disease and behavior is not fully understood (Chavez-Galan et al., 2015; Liu et al., 2021). To address this, we reanalyzed single-nucleus RNA sequencing (snRNA-seq) datasets from postmortem PD brains, uncovering a significant reduction of CD169^+^ BAMs in affected individuals (Prashant et al., 2024). This prompted us to examine whether loss of this BAM subset could contribute to brain dysfunction and behavioral phenotypes observed in neuropsychiatric and neurodegenerative conditions.

We hypothesized that CD169^+^ BAMs are essential for maintaining neural network stability and that their loss contributes to early, non-dopaminergic manifestations of Parkinsonian pathology. To test this, we selectively depleted CD169^+^ BAMs and evaluated their impact on behavior and olfactory circuitry. In the present study, we used CD169-DTR mice to selectively deplete CD169^+^ BAMs and assessed the resulting behavioral and histological changes (Saito et al., 2001; Miyake et al., 2007).

## Results

### CD169^+^ BAMs are reduced in patients with Parkinson’s disease

**Figure 1:**
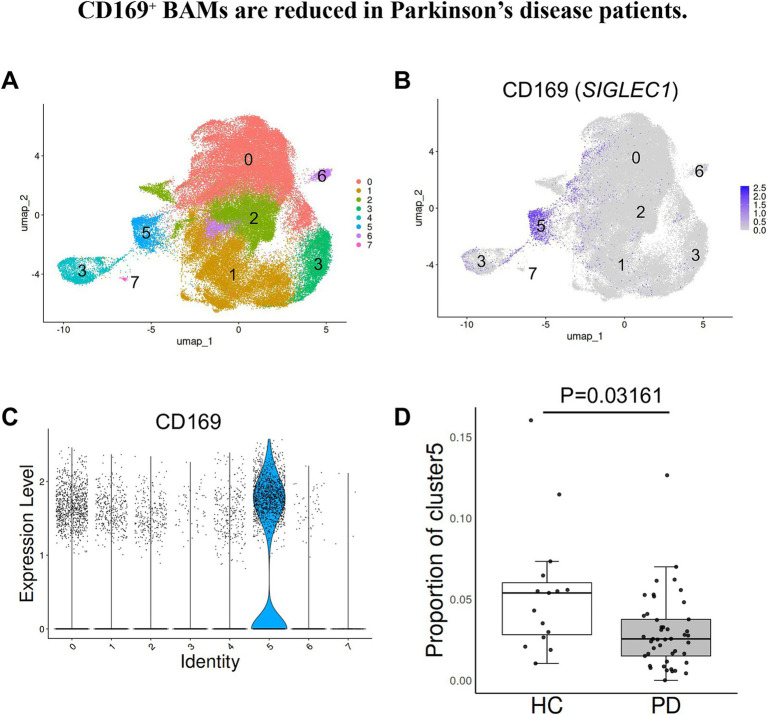
CD169^+^ BAMs are reduced in Parkinson’s disease patients. **(A)** UMAP plot of human brain macrophages obtained from 45 PD and 15 HC subjects. **(B)** The expression of CD169 on UMAP plot of human brain macrophages obtained from PD and HC subjects. **(C)** Violin plot showing CD169 expression in human brain macrophages obtained from PD and HC subjects. **(D)** The proportion of cluster 5 within brain macrophages from PD and HC subjects. Each dot represents each subject. Student’s *t*-test. A *p*-value of less than 0.05 was considered statistically significant.

Previous single-cell RNA sequencing studies have identified CD169 as a marker for specific subsets of border-associated macrophages (BAMs) in mice (Sun and Jiang, 2024). To assess the relevance of CD169^+^ BAMs in human neurodegenerative disorders, we reanalyzed publicly available single-nucleus RNA sequencing (snRNA-seq) data from brain tissue of individuals with Parkinson’s disease (PD) and healthy controls (Prashant et al., 2024). In our analysis, CD169 (*SIGLEC1*) expression was specifically enriched in cluster 5 (Figure 1A–C; Supplementary Figure 1) (Yeo et al., 2022). Notably, the proportion of cluster 5 cells within the brain macrophage population was significantly reduced in PD samples compared with controls, whereas no such reduction was observed in any of the other clusters (Figure 1D; Supplementary Figure 2). Given that this cluster represents CD169^+^ BAMs, our findings indicate that this BAM subset is selectively diminished in the context of PD. This reduction in human disease prompted us to investigate the functional role of CD169^+^ BAMs *in vivo*, with the hypothesis that their loss could destabilize neural networks and contribute to Parkinsonian motor and non-motor symptoms.

### CD169^+^ BAMs are specifically depleted in CD169-DTR transgenic mice

**Figure 2:**
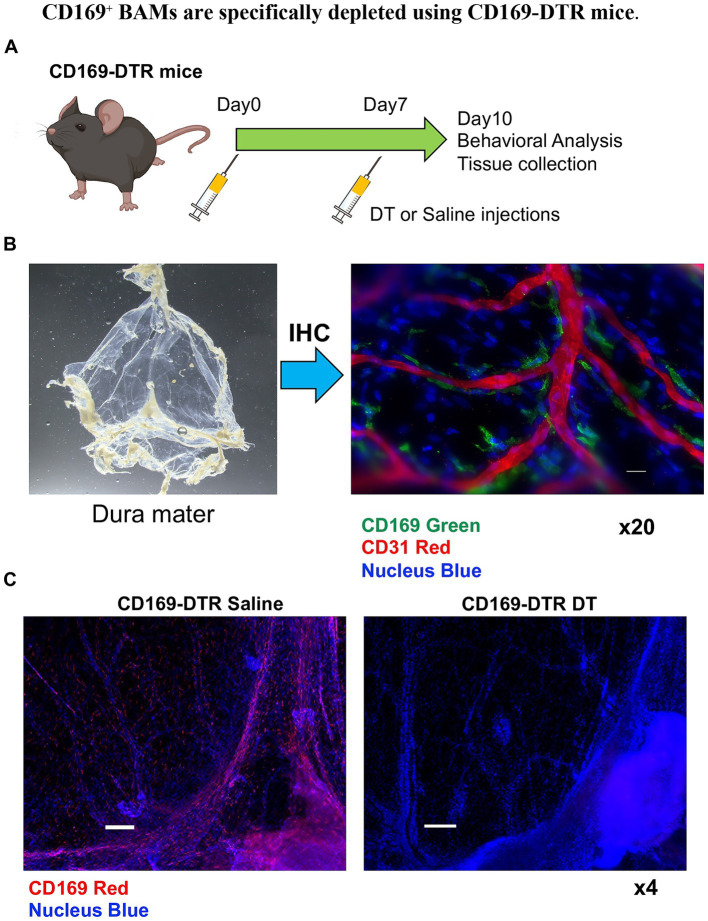
Localization and depletion of CD169^+^ border-associated macrophages (BAMs). **(A)** Experimental timeline showing DT administration and subsequent analysis. **(B)** Removed dura mater and immunohistochemical staining showing CD169^+^ BAMs (green) localized adjacent to CD31^+^ endothelial cells (red) in the dura mater, confirming their perivascular distribution. Nuclei are counterstained with Hoechst (blue). Scale bar, 20 μm. (*n* = 3 healthy mice). **(C)** Representative images showing effective depletion of CD169^+^ BAMs in CD169-DTR mice following diphtheria toxin (DT) administration. DT was administered intraperitoneally 1 day before the analysis. Immunohistochemical staining showing CD169^+^ BAMs (red) and nuclei counterstained with Hoechst (blue). (*n* = 3 CD169-DTR saline and *n* = 3 CD169 DTR DT). Scale bar, 200 μm. All experiments were independently replicated at least twice, and consistent results were obtained across replicates.

To investigate the *in vivo* function of CD169^+^ BAMs, we employed CD169-DTR transgenic mice, in which diphtheria toxin (DT) administration induces selective ablation of CD169-expressing cells (Saito et al., 2001; Miyake et al., 2007). To determine the behavioral consequences of CD169^+^ BAM loss, DT was administered twice at one-week intervals to CD169-DTR mice (Figure 2A). Immunohistochemistry (IHC) confirmed that CD169^+^ BAMs are located close to CD31^+^ blood vessels in the meninges and perivascular spaces, consistent with their known anatomical distribution and vascular-associated roles (Figure 2B) (Zhan et al., 2025). Administration of DT effectively depleted CD169^+^ BAMs in these mice, as confirmed by loss of CD169 immunoreactivity (Figure 2C).

### CD169^+^ BAM depletion induces motor and affective behavioral abnormalities

**Figure 3:**
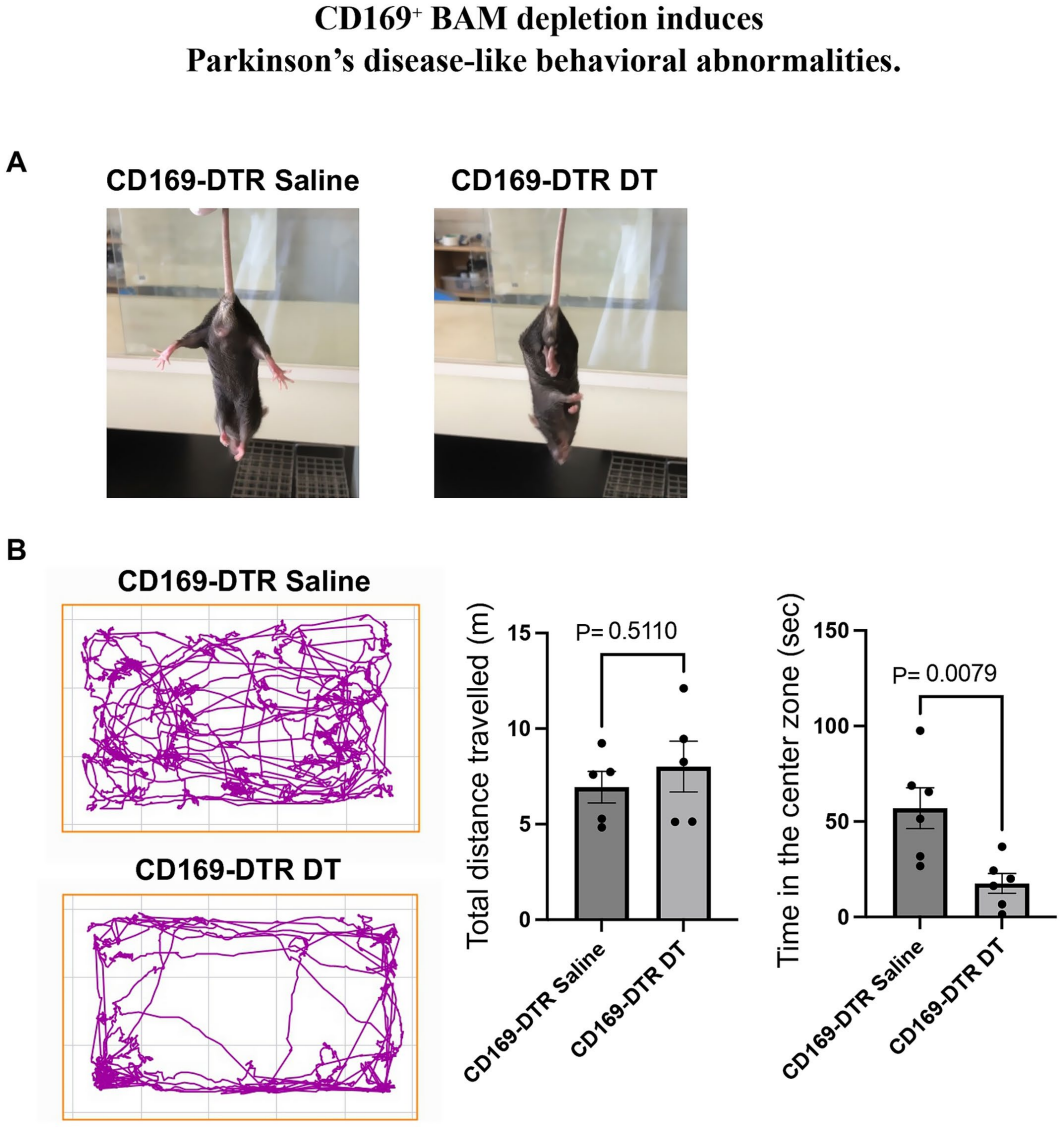
Behavioral abnormalities induced by CD169^+^ BAM depletion. **(A)** Representative images of mice displaying abnormal hindlimb clasping reflex in the CD169^+^ BAM-depleted group. (*n* = 3 CD169-DTR saline and *n* = 3 CD169 DTR DT). **(B)** Open field test results showing track plot (left) and total distance traveled, and time spent in the center zone (right). Data are presented as mean ± SEM. ^*^p < 0.05, Student’s *t*-test. (*n* = 5 CD169-DTR saline and *n* = 5 CD169 DTR DT). All experiments were independently replicated at least twice, and consistent results were obtained across replicates.

Remarkably, all CD169^+^ BAM-depleted mice developed tremors. Furthermore, abnormal hindlimb clasping was observed during the clasping reflex test, suggesting motor dysfunction (Figure 3A) (Zhao et al., 2017). Open field testing revealed that while total locomotor activity (distance traveled) was comparable between groups, CD169^+^ BAM-depleted mice spent significantly less time in the central zone, indicative of anxiety-like behavior (Figure 3B) (Seibenhener and Wooten, 2015). These phenotypes are reminiscent of clinical features observed in PD-like disorders.

### CD169^+^ BAM depletion disrupts the olfactory system without dopaminergic neuron loss

**Figure 4:**
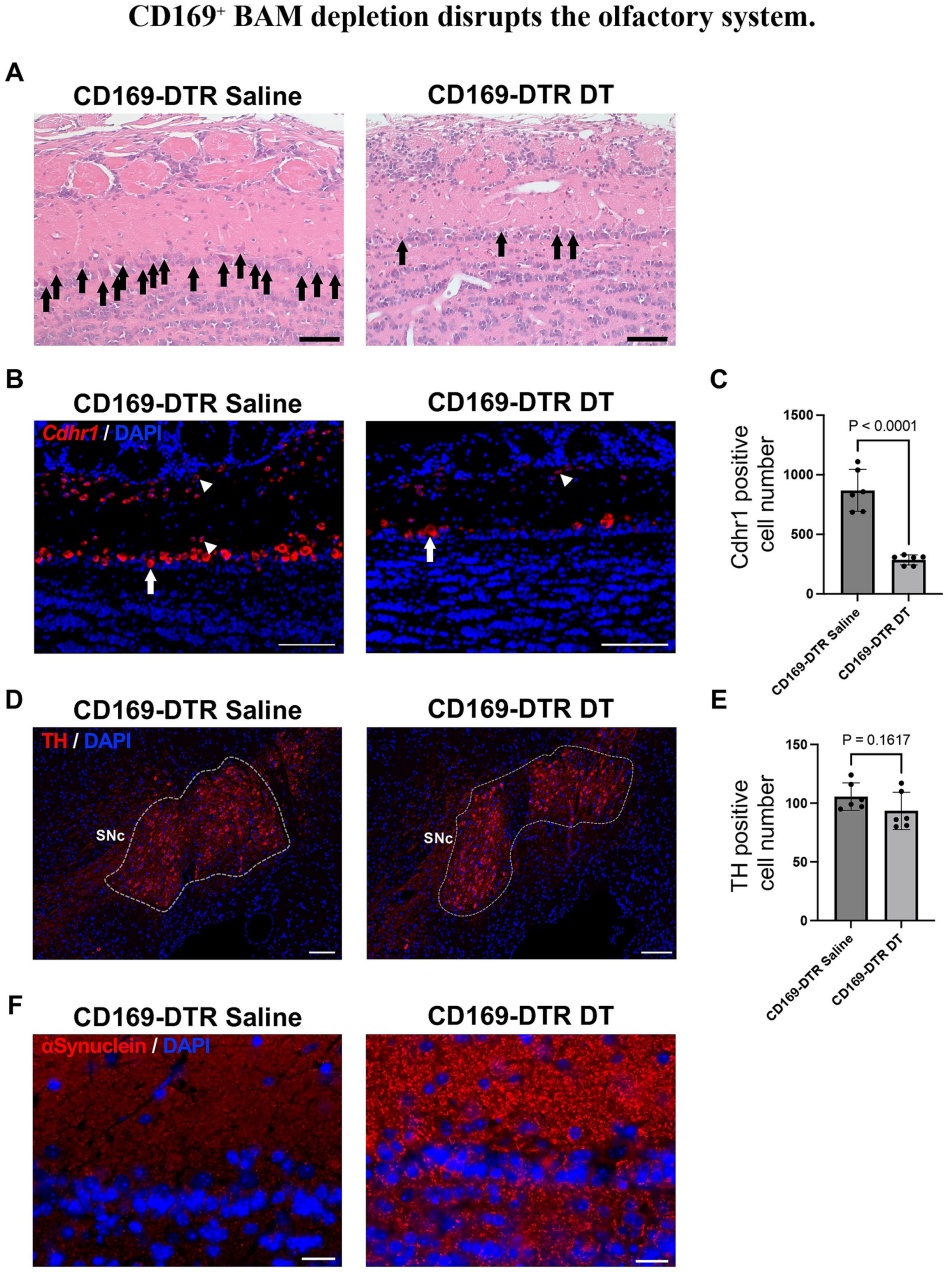
CD169^+^ BAM depletion disrupts the olfactory system. **(A)** Representative H&E stain images of the olfactory bulb from CD169^+^ BAM-depleted mice and controls. Arrows show cells that can be morphologically identified as mitral cells. Scale bars, 50 μm. (*n* = 3 CD169-DTR saline and *n* = 3 CD169 DTR DT). **(B)** Representative *in situ* hybridization images showing *Cdhr1* expression in the olfactory bulb of CD169^+^ BAM-depleted mice and controls. Nuclei are counterstained with DAPI (blue). Arrows indicate reaction on mitral cells, and arrow heads indicate reaction on tufted cells. Scale bars, 50 μm. (*n* = 3 CD169-DTR saline and *n* = 3 CD169 DTR DT). **(C)** Quantification of *Cdhr1* positive cells in the OB. Bars show the number of *Cdhr1* positive cells per section (mean ± SEM). Statistics: unpaired two-tailed *t*-test; *p*-values are indicated on the graph. (*n* = 6 CD169-DTR saline and *n* = 6 CD169 DTR DT). **(D)** Representative immunohistochemical staining images showing TH expression in the substantia nigra compacta (SNc) of CD169^+^ BAM-depleted mice and controls. Nuclei are counterstained with DAPI (blue). Scale bars, 100 μm. (*n* = 3 CD169-DTR saline and *n* = 3 CD169 DTR DT). **(E)** Quantification of TH positive cells in the OB. Bars show the number of *Cdhr1* positive cells per section (mean ± SEM). Statistics: unpaired two-tailed *t*-test; *p*-values are indicated on the graph. (*n* = 6 CD169-DTR saline and *n* = 6 CD169 DTR DT). **(F)** Representative immunohistochemical staining images showing α-synuclein expression in the olfactory bulb. Nuclei are counterstained with DAPI (blue). Scale bars, 20 μm. (*n* = 3 CD169-DTR saline and *n* = 3 CD169 DTR DT). All experiments were independently replicated at least twice, and consistent results were obtained across replicates.

To assess pathological changes in the brain of BAM-depleted mice, we next conducted histopathological examination by hematoxylin & eosin (HE). Notably, we observed a significant reduction in mitral cells in the olfactory bulb of CD169^+^ BAM-depleted mice (Figure 4A; Supplementary Figures 3-1, 3-2). Additionally, the expression of Cdhr1, a marker gene for both mitral and tufted cells, confirmed that not only mitral cells but also tufted cells were significantly reduced (Figures 4B,C). Reduction of mitral and tufted cells had further progressed 1 week later than the observed point, and they were almost lost (Supplementary Figure 4). Given that these neurons are the primary projection neurons of the olfactory bulb, it was revealed that CD169^+^ BAM-depleted mice exhibit disruption to the olfactory circuitry. Importantly, immunohistochemical analysis revealed no obvious loss of tyrosine hydroxylase (TH)-positive dopaminergic neurons in the substantia nigra compacta (SNc), indicating that the observed PD-like phenotypes occurred independently of classical dopaminergic neurodegeneration (Figures 4D,E). In the olfactory bulb of CD169^+^ BAM-depleted mice, enhanced reactivity of granular α-synuclein, the major causes of neural degeneration in PD, were observed (Figure 4F). However, Lewy body that are the inclusion bodies composed of α-synuclein were not observed (Figures 4A,F). These findings suggest that the loss of CD169^+^ BAMs selectively impairs the olfactory system, a deficit that may be linked to the emergence of Parkinson’s disease-like behavioral phenotypes in CD169^+^ BAM-depleted mice.

## Discussion

We show that loss of CD169^+^ BAMs induces tremors, abnormal hindlimb reflexes, and anxiety-like behavior without dopaminergic neuron loss. Moreover, we observed a marked reduction of mitral and tufted cells in the olfactory bulb—neurons that form the primary relay between the olfactory system and higher brain regions—suggesting disruption of olfactory-limbic connectivity (Pro-Sistiaga et al., 2007; Lane et al., 2020). These findings indicate that CD169^+^ BAMs are essential for maintaining neural network stability and motor function, and that their loss can trigger PD-like phenotypes in the absence of classical neurodegeneration.

In this study, we identify a previously unrecognized role of CD169^+^ border-associated macrophages (BAMs) in maintaining neural network stability and motor function. By reanalyzing single-nucleus RNA sequencing data from postmortem Parkinson’s disease (PD) brains, we found that a BAM subset characterized by *CD169* (*SIGLEC1*) expression is significantly reduced in PD patients compared with healthy controls (Prashant et al., 2024; Sun and Jiang, 2024; Yeo et al., 2022). Using a CD169-DTR mouse model, we demonstrate that selective ablation of this BAM subset induces tremors, abnormal hindlimb reflexes, and anxiety-like behavior, closely resembling key clinical features of PD-like disorders.

Our findings expand the current understanding of how immune cells at the brain’s borders contribute to neurodegenerative disorders’ symptoms. While microglia have been widely studied for their roles in synaptic regulation and neuroinflammation, BAMs—particularly defined subsets such as CD169^+^ BAMs—have received far less attention (Schonhoff et al., 2023; Vara-Perez and Movahedi, 2025; Silvin et al., 2023). The anatomical localization of CD169^+^ BAMs along blood vessels in the meninges and perivascular spaces positions them to regulate CNS-periphery communication, potentially influencing vascular permeability, cytokine signaling, and immune cell trafficking. Loss of this BAM subset could therefore trigger functional disturbances in brain circuits without directly causing neuronal loss.

One of the most notable findings in our study is the marked reduction of mitral and tufted cells in the olfactory bulb following CD169^+^ BAM depletion, accompanied by preserved tyrosine hydroxylase (TH)-positive dopaminergic neurons in the substantia nigra compacta. Given that olfactory dysfunction is one of the most well-established prodromal symptoms, this indicates that PD-like behavioral phenotypes can emerge independently of classical dopaminergic neurodegeneration, highlighting the potential importance of olfactory system dysfunction in early disease stages. The olfactory bulb is functionally connected to multiple limbic and motor-related regions, and disruption of its projection neurons may destabilize broader neural networks involved in motor control, emotional regulation, and sensory processing. Indeed, Respiration-induced periodic olfactory circuitry activity plays an important role in cognitive function in both humans and mice (Lieu et al., 2013). Given that olfactory deficits often precede motor symptoms in PD, our results suggest that BAM-mediated maintenance of olfactory circuitry could be critical in preventing early network instability. In the olfactory bulb of CD169^+^ BAM-depleted mice, a marked increase of granular α-synuclein was observed in the present study. Although it remains unclear whether α-synuclein causes neural death in the olfactory bulb, it is considered that CD169^+^ BAMs play an important role in the inhibition of α-synuclein misfolding.

These data also raise the possibility that immune dysregulation at the CNS borders is not merely a secondary consequence of neurodegeneration but may play a causative role in initiating disease-relevant phenotypes. The selective depletion of CD169^+^ BAMs in our model produced PD-like symptoms without overt directly causing neuronal death, providing a unique platform for dissecting how peripheral immune interfaces influence CNS circuitry in the prodromal phase of neurodegenerative disorders.

There are several limitations to our study. First, while we observed pronounced olfactory bulb pathology, the downstream network consequences were not directly measured using functional imaging or electrophysiology. Future work should aim to map the specific neural circuits altered by CD169^+^ BAM depletion. Second, although the CD169-DTR system allows for targeted cell depletion, CD169 expression is not entirely exclusive to BAMs, raising the possibility of effects on other myeloid populations. Finally, whether the reduction of CD169^+^ BAMs in human PD is a driver of disease or a byproduct of ongoing pathology remains to be determined.

In conclusion, our study identifies CD169^+^ BAMs as essential regulators of olfactory circuit integrity, motor function, and behavioral stability. Their loss leads to PD-like phenotypes in the absence of classical dopaminergic neurodegeneration, supporting a model in which brain-border immune cell dysfunction contributes to the initiation or progression of Parkinsonian pathology. This work establishes a novel animal model for studying neuroimmune mechanisms in PD and highlights BAMs as potential therapeutic targets for early intervention.

## Materials and methods

### Single-nucleus RNA sequencing data analysis

Single-nucleus RNA sequencing (snRNA-seq) data from brain tissues of individuals with Parkinson’s disease (PD) and matched healthy controls passing quality check by supplier were obtained from https://cellxgene.cziscience.com.^1^ The dataset was originally obtained from biobanks which operates to the highest ethical standards with appropriate informed consent from all participants as described in the source publication (NIH NeuroBioBank^2^ at the Mount Sinai School of Medicine, NIH NeuroBioBank at the Harvard Brain Tissue Resource Center, NIH NeuroBioBank at the University of Miami, the University of Miami Brain Endowment Bank^3^ and the University of Miami Udall Center of Excellence for Parkinson’s Disease Research^4^) (Chavez-Galan et al., 2015). We adhered to all data usage conditions and access requirements specified in the dataset metadata and by the originating repository (AMP PD Knowledge Platform). No attempts were made to re-identify individual donors.

The analysis was conducted using only sample data obtained from male subjects. At first, cells annotated as “central nervous system macrophage” by the supplier were isolated and converted to seurat object from AnnData. Then, UMAP dimension reduction and identification of genes expressed in each cluster by differential expression analysis were performed using the R package Seurat (Version 4.3.3) (Hao et al., 2021).

### Animals

C57BL/6 mice were purchased from SLC (Shizuoka, Japan). Homozygous CD169^DTR/DTR^ knock-in (Siglec1^tm1(HBEGF)Mtka^) mice were sourced from Riken Bio Resource Centre (Yokohama, Kanagawa, Japan) (Saito et al., 2001; Miyake et al., 2007). Heterozygous CD169^+/DTR^ mice were generated by crosses of CD169^DTR/DTR^ males with C57BL/6 background females and maintained under specific pathogen-free (SPF) conditions. All animals were housed in temperature- and humidity-controlled rooms on a 12-h light/dark cycle with ad libitum access to food and water. Male mice aged 6–10 weeks were used for all experiments. All procedures were approved by the Animal Care and Use Committee of Gifu University (Approval No. AG-P-N—20240063) and were conducted in accordance with institutional and national guidelines for animal research.

### CD169^+^ cell depletion

To selectively ablate CD169^+^ border-associated macrophages (BAMs), CD169-DTR mouse received 0.5 μg of diphtheria toxin (DT; Fuji Film, 048-34371) via intraperitoneal injections. Injections were performed twice at a one-week interval to ensure sustained depletion of CD169^+^ cells. Control mice received saline injections.

### Immunohistochemistry

Mice were perfused transcardially with PBS followed by 4% paraformaldehyde in PBS, and the brain was removed and routinely embedded in paraffin. For the preparation of paraffin sections, sections (3.75 μm) were cut with a microtome and mounted on MAS-coated slides (Matsunami Glass Ind., Ltd., Osaka, Japan). Paraffin sections were rehydrated through a xylene-ethanol gradient and subjected to antigen retrieval treatment with immunosaver (New EM Co., Ltd., Tokyo, Japan) according to the manufacturer’s instructions. Sections were incubated with primary antibodies against CD169 (Biolegend, Catalog number: 142408), CD31 (R and D Systems, Catalog number: AF3628), TH (Abcam, ab113), and α-synuclein (Cell Signaling Technology, Catalog number: 4179) overnight at 4 °C. After washing, appropriate fluorescently labeled secondary antibodies (Jackson ImmunoReseach Lab) were applied. Images were acquired using a BZ-X810 microscope (KEYENCE, Osaka).

### *In situ* hybridization

Paraffin sections of mouse brain were prepared according to the method described above. After rehydration with a xylene-ethanol gradient, sections were treated with 5 μg/mL proteinase-K in PBS for 30 min at 37 °C. Then, acetylation was performed with 0.1 M triethanolamine buffer (pH 8.0) containing 0.25% (v/v) acetic anhydride for 10 min at RT. After that, sections were dehydrated with an ethanol gradient and dried completely. Dried sections were incubated with hybridization buffer containing 50% (v/v) formamide, 10% dextran sulfate sodium, 0.15 M NaCl, 0.015 M sodium citrate, 1% blocking reagent (Roche Diagnostics GmbH, Mannheim, Germany), 0.01% sodium n-dodecanoyl sarcosinate, 0.01% sodium dodecyl sulfate (SDS), and 2 ng/μL digoxigenin (DIG)-tagged RNA probe targeting *Cdhr1* messenger RNA for 18 h at 45 °C. The probe was synthesized from an amplicon of reverse transcription polymerase chain reaction (RT-PCR) with forward primer (5′-ACACACAGGGGAAATTAGGCTCAAG-3′) and reverse primer (5′-GTGATTGTGGGGTCTAGAGTTGGTG-3′) using DIG RNA Labeling Kit (Roche Diagnostics GmbH) according to the manufacturer’s instructions. After hybridization, sections were rinsed with buffer containing 50% (v/v) formamide, 0.15 M NaCl, 0.015 M sodium citrate, and 0.01% sodium n-dodecanoylsarcosinate for 15 min twice at 45°. Then, RNase treatment was performed with 0.02 mg/mL RNase-A (Roche Diagnostics GmbH) dissolved in NTE buffer (100 mM NaCl, 10 mM tris hydroxymethyl aminomethane (Tris), and 1 mM ethylenediaminetetraacetic acid (EDTA) for 15 min at 37 °C). After RNase treatment, peroxidase-conjugated anti-DIG sheep polyclonal antibody (Roche Diagnostics GmbH, Catalog number: 11207733910) diluted 200 times was applied for 1 h at 37 °C. The following colorization of signals was conducted by tyramide signal amplification (TSA). To illustrate, 100 mM borate buffer containing 5 μM Alexa-Fluor 594 conjugated tyramide (Lumiprobe Limited, Wan Chai, Hong Kong, Catalog number: 3804), 2% dextran sulfate sodium, and 0.015% H_2_O_2_ was applied to sections for 15 min at RT. Images were acquired using a BZ-X810 microscope (KEYENCE, Osaka).

### Hematoxylin and eosin staining

To assess morphological changes, brain sections were stained with H&E following standard protocols. Briefly, paraffin sections were mounted on slides, rehydrated through a xylene-ethanol gradient, stained with hematoxylin, differentiated, and counterstained with eosin. Slides were dehydrated and mounted with coverslips. Images were captured under a brightfield microscope.

### Behavioral assessments

Behavioral experiments were conducted in a dedicated behavioral testing room under consistent lighting and noise conditions. Clasping test: Mice were suspended by the tail for 15 s, and hindlimb position was recorded. Abnormal clasping was defined as hindlimbs drawn toward the abdomen and sustained for more than 3 s (Lieu et al., 2013). Open field test: Mice were placed in the center of a 15 cm × 25 cm open field arena and allowed to explore freely for 5 min. Movement was recorded and analyzed using ANY-maze software (Stoelting) (Seibenhener and Wooten, 2015). Total distance traveled and time spent in the center zone were measured to assess locomotor activity and anxiety-like behavior.

### Cell counting on tissue sections

Cell counts were performed on tissue sections. Cell bodies exhibiting reactivity for *Cdhr1* or TH in the olfactory bulb or SNc, respectively, were identified under a fluorescent microscope. For each specimen, the number of positive cell bodies was manually counted.

### Quantification and statistical analysis

Quantitative data are presented as mean ± standard error of the mean (SEM). Statistical analyses were performed using GraphPad Prism 9. Comparisons between two groups were made using unpaired two-tailed Student’s *t*-tests. A *p*-value of <0.05 was considered statistically significant.

## Data Availability

The raw data supporting the conclusions of this article will be made available by the authors, without undue reservation.

## Footnotes

1
https://datasets.cellxgene.cziscience.com/5c56796c-849f-4919-a680-15f1a3adcd91.h5ad

2
https://neurobiobank.nih.gov/

3
https://med.miami.edu/programs/brain-endowment-bank

4
https://udall.umn.edu/

## Supplementary material

SUPPLEMENTARY FIGURE 1 Dot plot showing expression of the top 10 genes with the greatest differences in expression levels between clusters.

SUPPLEMENTARY FIGURE 2 The proportion of all clusters other than cluster 5 within brain macrophages from PD and HC subjects. Each dot represents each subject. Student’s *t*-test.

SUPPLEMENTARY FIGURE 3 Representative H&E stain images of the cerebral cortex, hippocampus, striatum, amygdala, cerebellum, and medulla oblongata from CD169⁺ BAM-depleted mice and controls. Scale bars, 100 μm. (*n* = 3 CD169-DTR saline and *n* = 3 CD169 DTR DT). All experiments were independently replicated at least twice, and consistent results were obtained across replicates.

SUPPLEMENTARY FIGURE 4 **(A)** Representative *in situ* hybridization images showing *Cdhr1* expression in the olfactory bulb of CD169⁺ BAM-depleted mice and controls at one week later than observed point. Nuclei are counterstained with DAPI (blue). Arrows indicate reaction on mitral cells, and arrow heads indicate reaction on tufted cells. Scale bars, 100 μm. (*n* = 3 CD169-DTR saline and *n* = 3 CD169 DTR DT). **(B)** Quantification of *Cdhr1* positive cells in the olfactory bulb. Bars show the number of *Cdhr1* positive cells per section (mean ± SEM). Statistics: unpaired two-tailed *t*-test; *p*-values are indicated on the graph. (*n* = 6 CD169-DTR saline and *n* = 6 CD169 DTR DT). All experiments were independently replicated at least twice, and consistent results were obtained across replicates.
