# Supplementary figures and images for: Depletion of CD169+ border-associated macrophages induces Parkinson’s disease-like behavior
Source: Front Neurosci. 2025 Dec 4;19:1688394. doi: 10.3389/fnins.2025.1688394 (PMC12713354; doi:10.3389/fnins.2025.1688394)

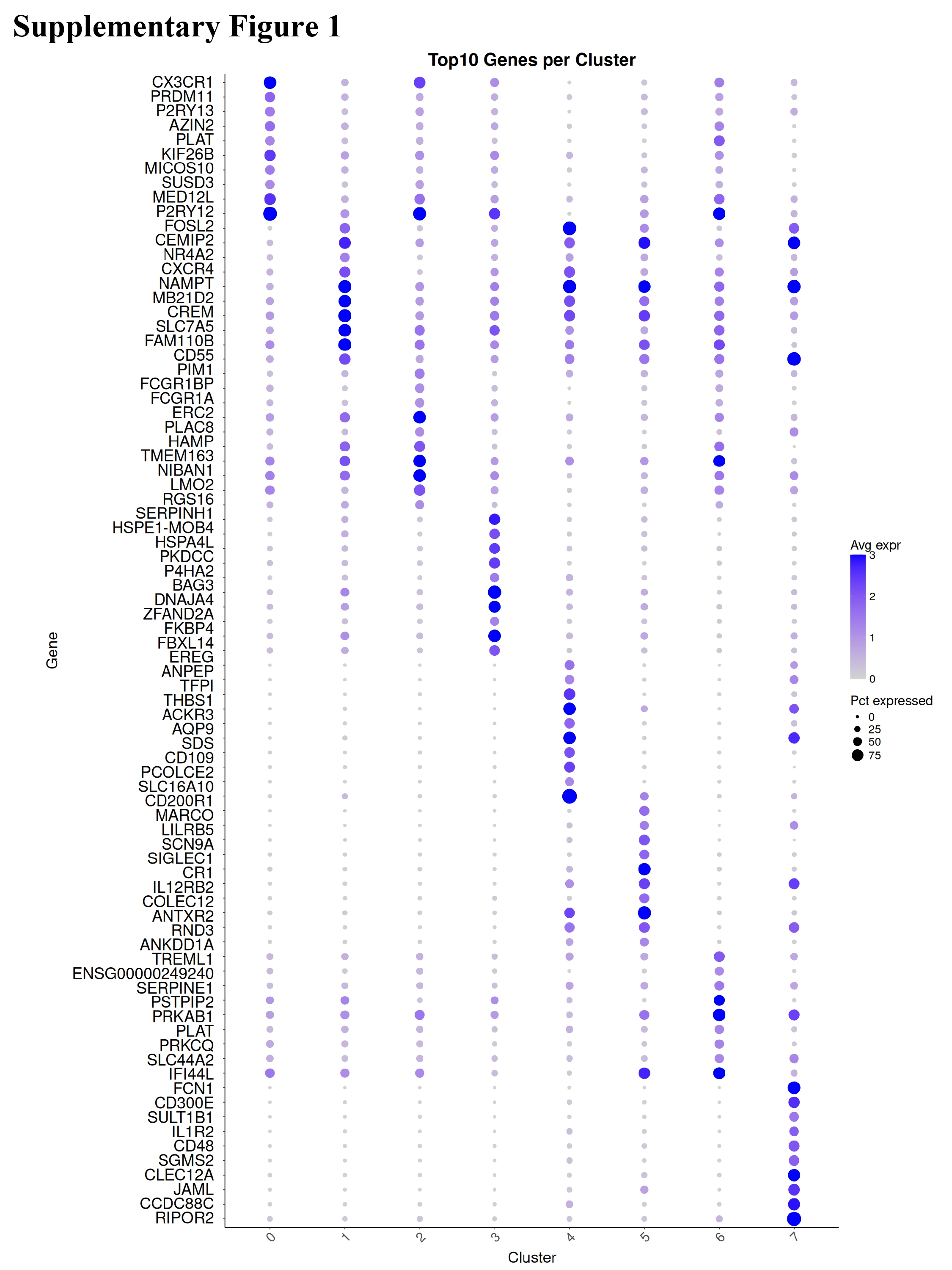

Supplement: SUPPLEMENTARY FIGURE 1 — Dot plot showing expression of the top 10 genes with the greatest differences in expression levels between clusters. [file Supplementary_file_1.zip › Supplementary figure 1.JPEG]

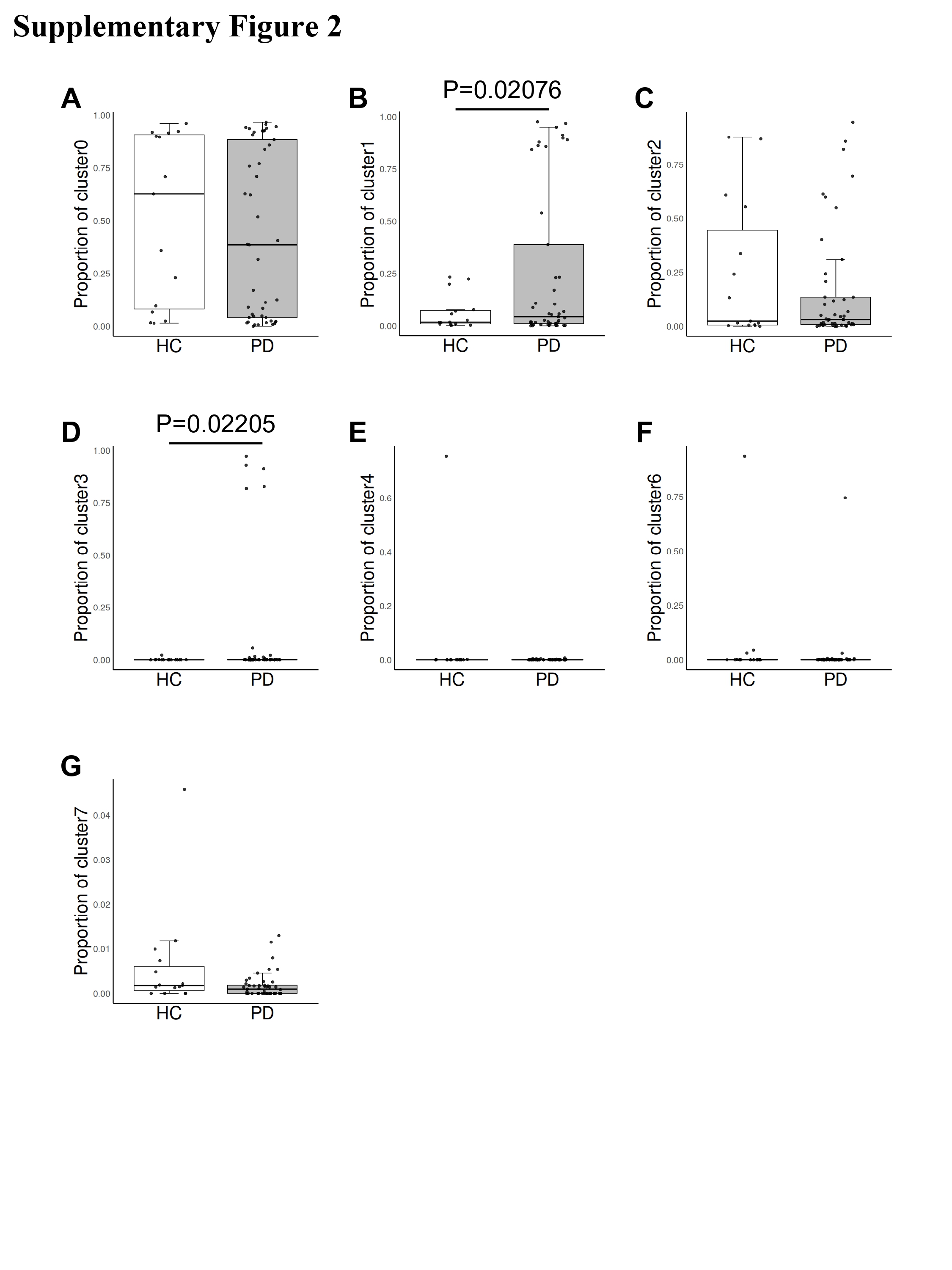

Supplement: SUPPLEMENTARY FIGURE 1 — Dot plot showing expression of the top 10 genes with the greatest differences in expression levels between clusters. [file Supplementary_file_1.zip › Supplementary figure 2.JPEG]

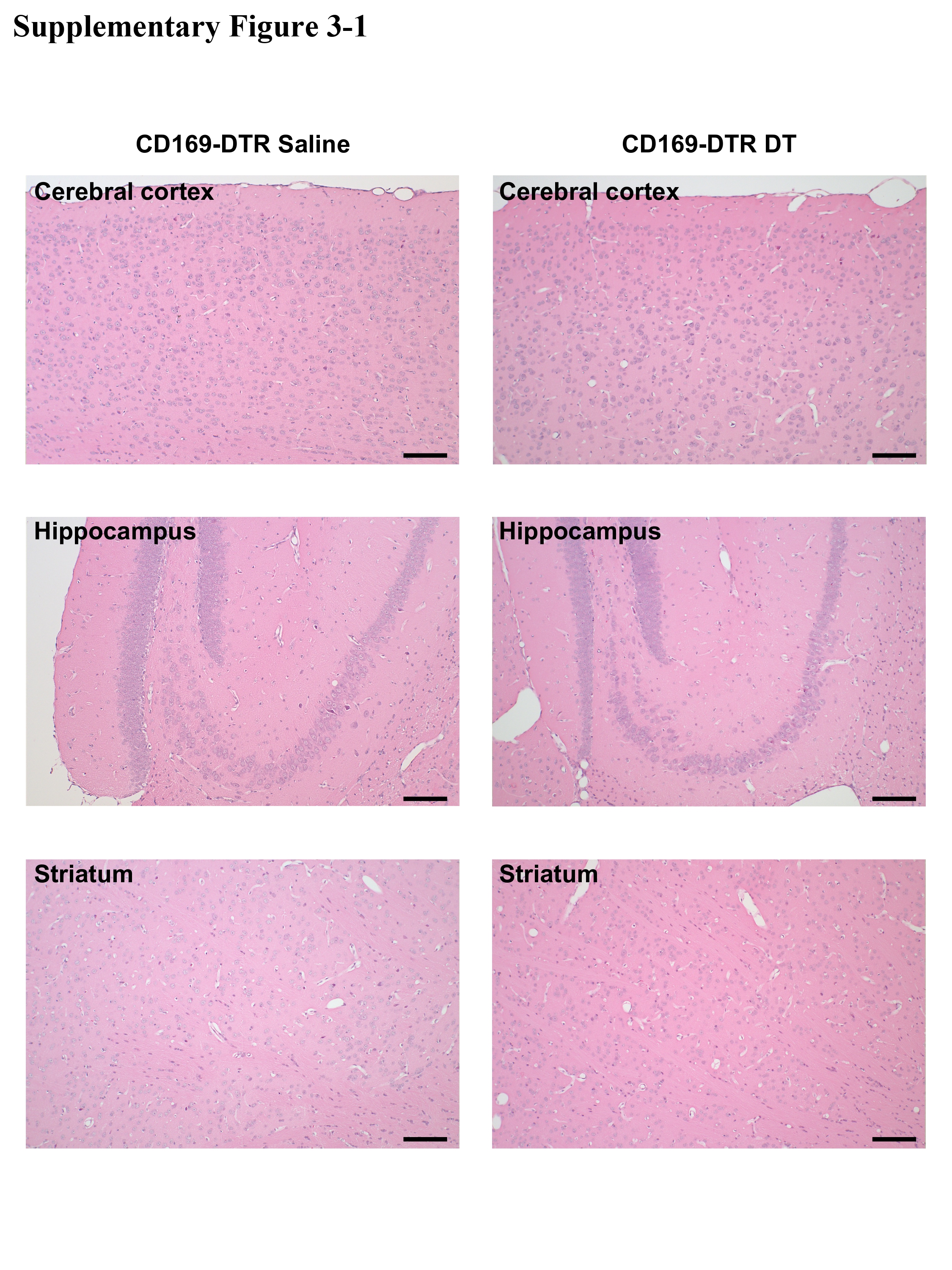

Supplement: SUPPLEMENTARY FIGURE 1 — Dot plot showing expression of the top 10 genes with the greatest differences in expression levels between clusters. [file Supplementary_file_1.zip › Supplementary figure 3-1.JPEG]

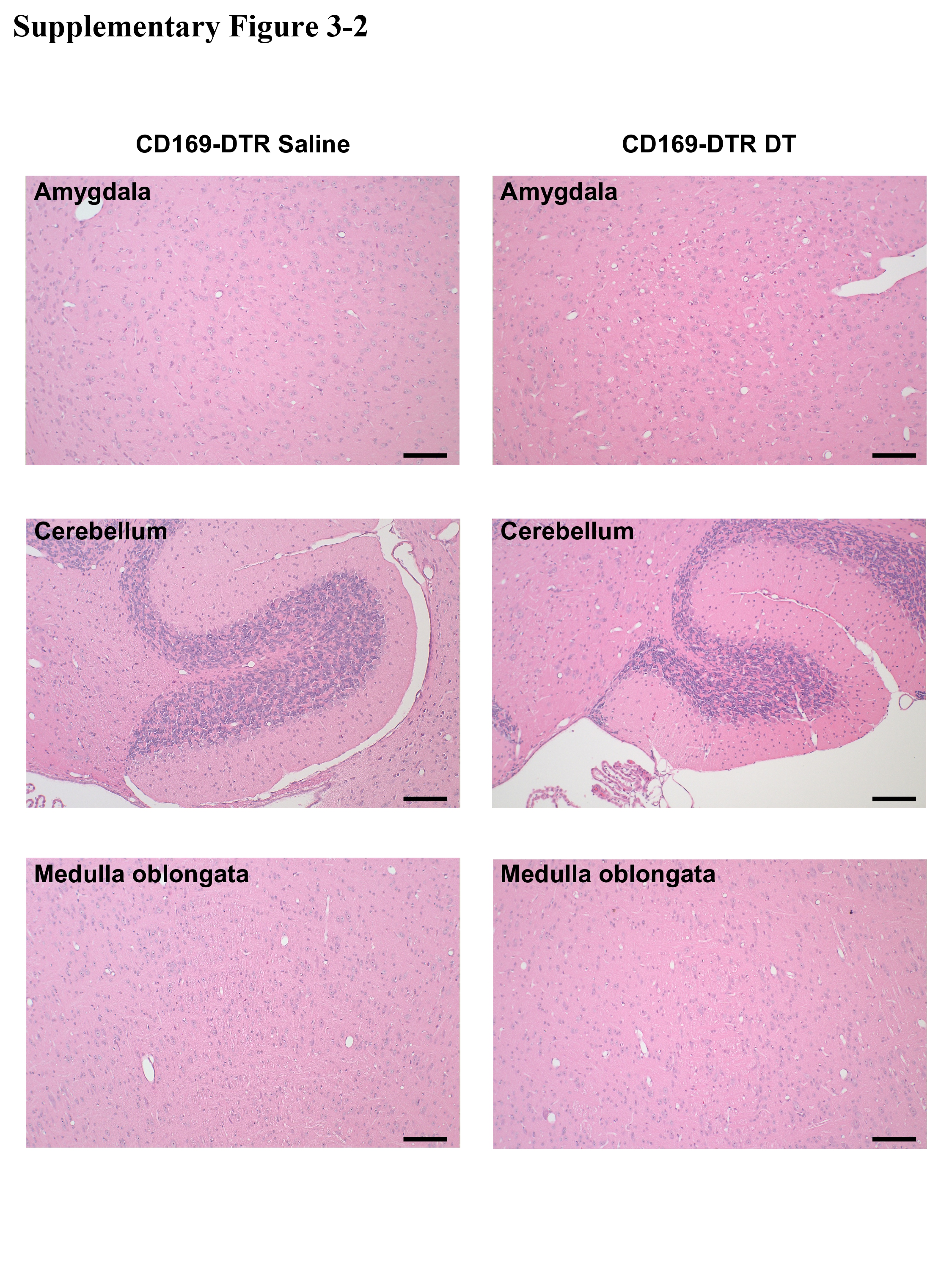

Supplement: SUPPLEMENTARY FIGURE 1 — Dot plot showing expression of the top 10 genes with the greatest differences in expression levels between clusters. [file Supplementary_file_1.zip › Supplementary figure 3-2.JPEG]

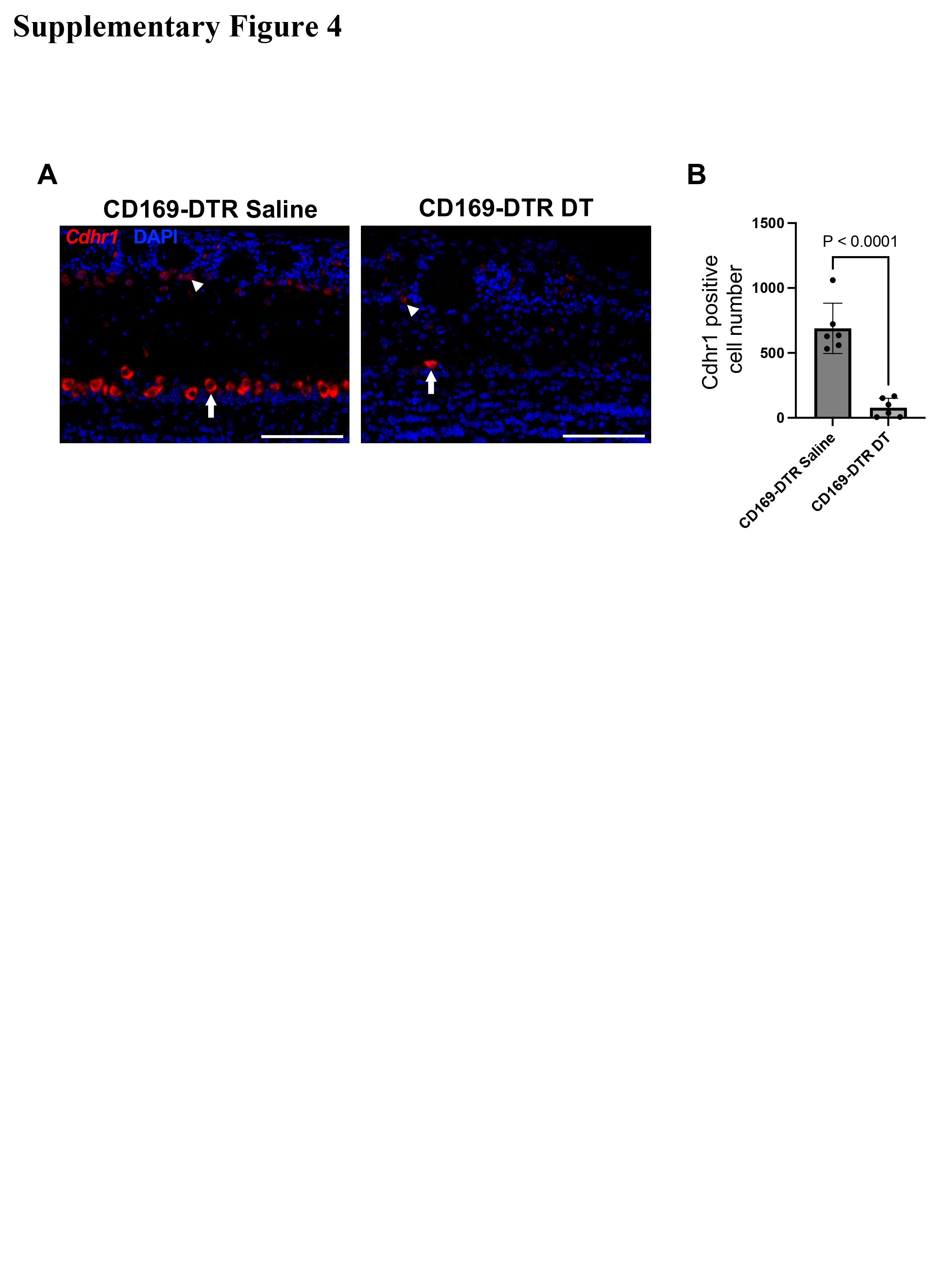

Supplement: SUPPLEMENTARY FIGURE 1 — Dot plot showing expression of the top 10 genes with the greatest differences in expression levels between clusters. [file Supplementary_file_1.zip › Supplementary figure 4.JPEG]
